# Effects of ultrasonication time on stability, dynamic viscosity, and pumping power management of MWCNT-water nanofluid: an experimental study

**DOI:** 10.1038/s41598-020-71978-9

**Published:** 2020-09-16

**Authors:** Amin Asadi, Ibrahim M. Alarifi

**Affiliations:** 1 Institute of Research and Development, Duy Tan University, Da Nang, 550000 Vietnam; 2 Faculty of Natural Sciences, Duy Tan University, Da Nang, 550000 Vietnam; 3 Department of Mechanical and Industrial Engineering, College of Engineering, Majmaah University, Al-Majmaah, 11952 Riyadh Saudi Arabia; 4 Engineering and Applied Science Research Center, Majmaah University, Al-Majmaah, 11952 Riyadh Saudi Arabia

**Keywords:** Chemical engineering, Mechanical engineering

## Abstract

It is known that ultrasonication has a certain effect on thermophysical properties and heat transfer of nanofluids. The present study is the continuation of the authors’ previous research on the effects of ultrasonication on the thermophysical properties of Multi-Walled Carbon Nanotubes (MWCNTs)-water nanofluid. Investigating the effects of ultrasonication time on samples’ stability, rheological properties, and pumping power of a water-based nanofluid containing MWCNTs nanoparticle is the main objective of the present study. The two-step method has been employed to prepared the samples. Moreover, a probe-type ultrasonic device has been used, and different ultrasonication times have been applied. The samples’ stability is investigated in different periods. The results revealed that prolonging the ultrasonication time to 60 min leads to improving the samples’ stability while prolonging ultrasonication time to higher than 60 min resulted in deteriorating the stability. As for dynamic viscosity, it is observed that increasing ultrasonication time to 60 min leads to decreasing the dynamic viscosity of the samples. As for pumping power, it is observed that the maximum increase in fanning friction factor ratio is less than 3%, which shows that adding MWCNTs to water does not impose a considerable penalty in the required energy for pumping power.

## Introduction

There is no denying the fact that introducing nanofluids (NFs), which are suspensions of nano-sized particles into the conventional working fluids, by Choi and Eastman^1^ in 1995 has opened new doors to improve the heat transfer performance in different applications. After this pioneering study by Choi and Eastman^1^, many researchers investigated the stability and thermophysical properties^2–4^, heat transfer^5–11^, applications of artificial intelligence in predicting the thermophysical properties of nanofluids^12–16^, and applications of various NFs^17–21^. It is known that thermophysical properties of each fluid play an important role in heat transfer performance of the fluids: viscosity directly affected the pumping power and the pressure loss, thermal conductivity indicates the heat transfer effectiveness of the fluid, and specific heat capacity shows the capability of the fluid in storing and moving heat away. Adding nano-sized particles to conventional coolants (i.e., water, oil, ethylene glycol, and so forth) leads to improving the thermal properties of the conventional coolants, which is highly desirable from the heat transfer point of view, although it causes some penalty in pumping power and pressure loss, which is not desirable from the energy management point of view.

It is accepted by all researchers that the most crucial step towards conducting experimental investigations using NFs is the sample preparation step. Suspending the nanoparticles (NPs) into the base fluid with the minimum sedimentation and agglomeration of the particles is a long-lasting challenge in employing NFs in practical applications. NPs naturally tend to sediment and agglomerate, which leads to having a non-uniform suspension. Uniform distribution of NPs into the base fluid leads to having higher thermal conductivity, and as a result, higher heat transfer performance while the non-uniform distribution of the NPs leads to deteriorating the thermophysical properties of the NFs or even leads to having lower thermophysical properties compared to the base fluids. Thus, the preparation step is a critical step in conducting an experimental study. Various methods and techniques have been applied so far by researchers to prepare long-time stable samples (at least two weeks) with minimum sedimentation and agglomeration. Generally, the samples are prepared using single-step or two-step methods. The simultaneous preparation and dispersion of nanomaterials into the base fluid is the procedure of the single-step method while in the two-step method, the nanomaterials are produced first, and then they disperse in the base fluid applying various mixing techniques^22^. Although single-step method leads to having more stable NFs, there are various disadvantages as well, such as limited application of the prepared samples which is limited to be used in low vapor pressure process and managing the size and structure of the NPs is challenging. As for the two-step method, which is a widely used method in literature, the main advantage is that it is the most economical method in preparing the samples^22^.

It is known that applying ultrasonication plays a crucial role in the two-step method. It is indicated in the literature that ultrasonication results in breaking down the large agglomeration of NPs into the smaller-sized agglomeration which results in having a more stable suspension with uniform distribution of NPs into the base fluid^23–26^. Literature showed that ultrasonication time, power, and type of ultrasonication device has certain effect on stability^27,28^ and thermophysical properties of NFs^29–31^. It is indicated that ultrasonic probe devices are more effective than that of the ultrasonic bath devices^27,32^, and prolonging the ultrasonication time leads to improving the thermal conductivity^33^ and decreasing the dynamic viscosity^34^ of the NFs. However, there are some references which reported an optimum ultrasonication time in which prolonging the ultrasonication time to higher than those values leads to deteriorating the stability and thermophysical properties of the NFs^35–37^. Thus, finding the optimum sonication time is of paramount importance in the preparation of NFs to achieve the best stability and thermophysical properties.

Based on what has been discussed thus far, it is evident that ultrasonication time has certain effects on the stability and thermophysical properties of NFs. Moreover, it is known that thermophysical properties affected the heat transfer performance and pressure loss in a system in which NFs employed as a heat transfer fluid. The present paper is the continuation of the authors' previous study on the effects of ultrasonication time on stability and thermal conductivity of the nanofluid^38^. In the present study, the effects of ultrasonication time on dynamic viscosity and pressure loss of MWCNT-water in three different solid concentrations of 0.1, 0.3, and 0.5 vol% and different temperatures ranging from 25 to 60 °C will be studied. The optimum ultrasonication time which leads to having the minimum dynamic viscosity will be found, and the dynamic viscosity of NF will be measured in different studied temperatures and solid concentrations. Finally, in order to show how adding the MWCNT to water affects the required pumping power of the system, the pressure loss has been studied.

## Materials and methods

### Sample preparation

As it is discussed in the introduction, there are two commonly used methods to prepare the NFs’ samples; single-step and two-step methods. The advantages of using the two-step method have been discussed in the introduction. Thus, in the present study, the two-step method has been employed to prepare the samples in three different solid concentrations of 0.1, 0.2, and 0.5 vol% containing MWCNT (US Research Nanomaterials Inc., USA) NPs. The detailed information of MWCNT NP has been presented in Tab. I, under the supporting information section. Moreover, the TEM image of the MWCNT, which has been provided by the manufacturer, has been presented in Fig. I (under the supporting information section).

A schematic view of the process of the two-step method to prepare the NFs has been depicted in Fig. II (under the supporting information section). First, the NPs were added to the base fluid. Employing a mechanical stirrer for 2 h, the NPs have been dispersed into the base fluid. In order to break down the large agglomeration of the NPs into the smaller size clusters or even single particle, a probe-type ultrasonic device (Hielscher UP400S, 400 W, 24 kHz, Germany) was employed. In order to study the effects of ultrasonication on the stability and rheological properties of the NF, different ultrasonication times of 10, 20, 40, 60, 70, 75, and 80 min were applied. The effectiveness of the ultrasonication process has been proven in the literature^39–42^. However, optimum ultrasonication time is still challenging. This way, the optimum ultrasonication time, which results in having long-time stable NF with the minimum sedimentation, has been investigated, and the results will be presented in the following sections.

### Dynamic viscosity measurement

**Figure 1:**
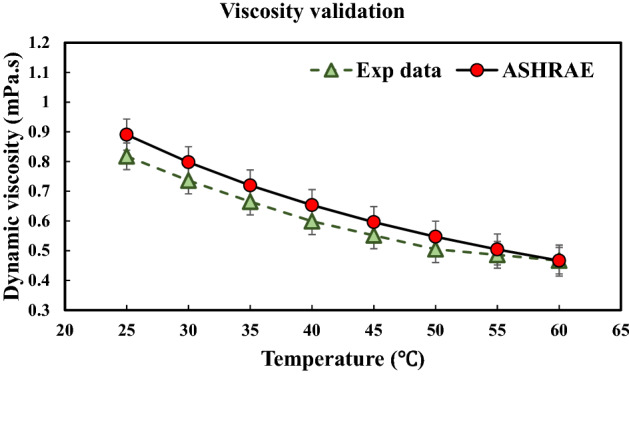
Results of the calibration test.

Searching the literature revealed that the most accurate and widely used device to measure the dynamic viscosity of different NFs is the Brookfield cone and plate viscometer manufactured by Brookfield Engineering Co., USA. In the present study, the CAP2000 + L viscometer has been employed to measure the dynamic viscosity of the MWCNT-water NF over different ranges of temperatures and solid concentrations. It should be noted that this series of viscometers are medium to high shear rate devices. It is interesting to know that the viscometer is equipped with an integrated temperature control system to control the temperature of the samples. The viscometer can operate in a wide range of rotational speeds ranging from 5 to 1,000 *RPM*, and the temperature control of the samples is possible between 5 to 75 °C with the accuracy of ± 0.5 °C. The calibration of the viscometer has been scrutinized before starting the experiments by measuring the dynamic viscosity of water and comparing the results whit those available in the ASHRAE handbook^43^. Figure 1 presents the results of the calibration test. As can be seen, there is a negligible difference (less than 8%) between the measured data and the data presented in the ASHRAE handbook^43^. Thus, it can be concluded that the viscometer is calibrated, and the measured data are reliable.

### Stability analysis

It is known that the stability of NFs is a critical and challenging issue in implementing NFs in practical applications. Different characterization analysis can be employed to determine the stability of NFs, such as:

- Electron Microscopy; Scanning electron microscopy (SEM), and transmission electron microscopy (TEM)
- Sedimentation technique
- Spectral analysis
- Zeta potential
- Dynamic light scattering (DLS).

In the present study, visual sedimentation and zeta potential analysis have been employed to investigate the effects of ultrasonication time on the stability of the samples over 30 days after the preparation of the samples. These two methods have been widely used in the literature by different researchers^33,44,45^. Figure III (under the supporting information section) shows the results of the visual observation of the samples in different periods after preparing the samples. It can be seen that the samples showed no sedimentation on the first day of preparation. The samples also showed good stability on the 5th day except for the sample, which was subjected to 80 min ultrasonication. After 10 days of preparation, the samples subjected to 75 and 80 min ultrasonication showed considerable sedimentation, and the sample subjected to 70 min ultrasonication showed negligible sedimentation. The rest of the samples are in good stability. As for the 30th day, massive sedimentation has been observed for the samples subjected to more than 70 min ultrasonication, but the rest of the samples are in good stability. From the visual observation, it can be concluded that prolonging the ultrasonication time to more than 60 min leads to deteriorating the stability of the samples. The results of the further accurate investigation by conducting zeta potential analysis on the stability of the samples subjected to different ultrasonication time will be presented in the following.

**Figure 2:**
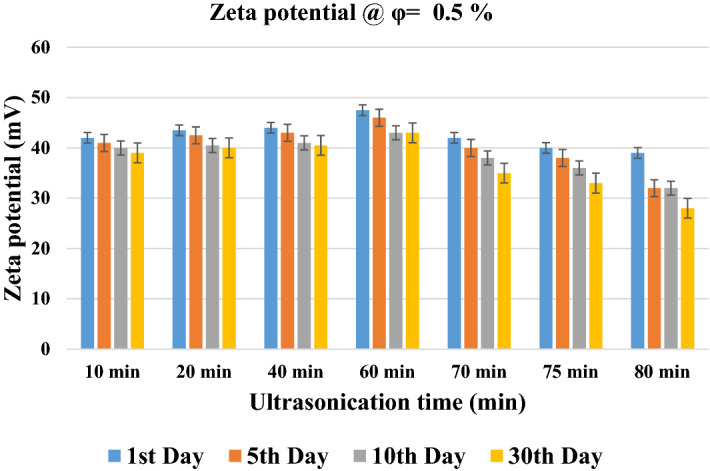
Zeta potential concerning ultrasonication time at different times after sample preparation.

Zeta potential is a quantitative analysis that measures the interfacial potential between the dispersion medium (base fluid) and the thin layer attached to the surface of the NPs. The unit of the zeta potential (ζ) is *mV*, and higher values of zeta potential show better stability of the suspensions. It should be noted that the samples with the zeta potential lower than 30 mV consider as limited stability (unacceptable stability), between 30 to 60 mV considers as physical stability (good/moderately acceptable stability), and the samples with the zeta potential higher than 60 mV consider as excellent stability^27,44^. Figure 2 presents the results of zeta potential with respect to ultrasonication time at the solid concentration of 0.5 vol%, which is the highest studied solid concentration. As can be seen, increasing the ultrasonication time from 20 to 60 min resulted in increasing the value of zeta potential, which means higher stability. However, a further increase in ultrasonication time (higher than 60 min) leads to deteriorating the stability of the NF. The trend is approximately similar in different periods of time at which the zeta potential analysis has been performed. It can also be seen that the sample subjected to 60 min ultrasonication possesses the best stability even after 30 days of preparation. Thus, it can be concluded that 60 min ultrasonication is the optimum time in which the best stability can be achieved for the studied NF.

## Results and discussion

In the following sections, first of all, the rheological behavior of the studied nanofluid will be investigated over a different range of temperatures, solid concentrations, and shear rates. Then, the effects of ultrasonication time on the variations of dynamic viscosity will be presented and discussed. Moreover, the results of the dynamic viscosity measurement in different temperatures and solid concentrations, at the optimum ultrasonication time will be presented and discussed. Apart from that, the effects of adding MWCNT to the water on the pressure loss will be studied.

### Rheological behavior

**Figure 3:**
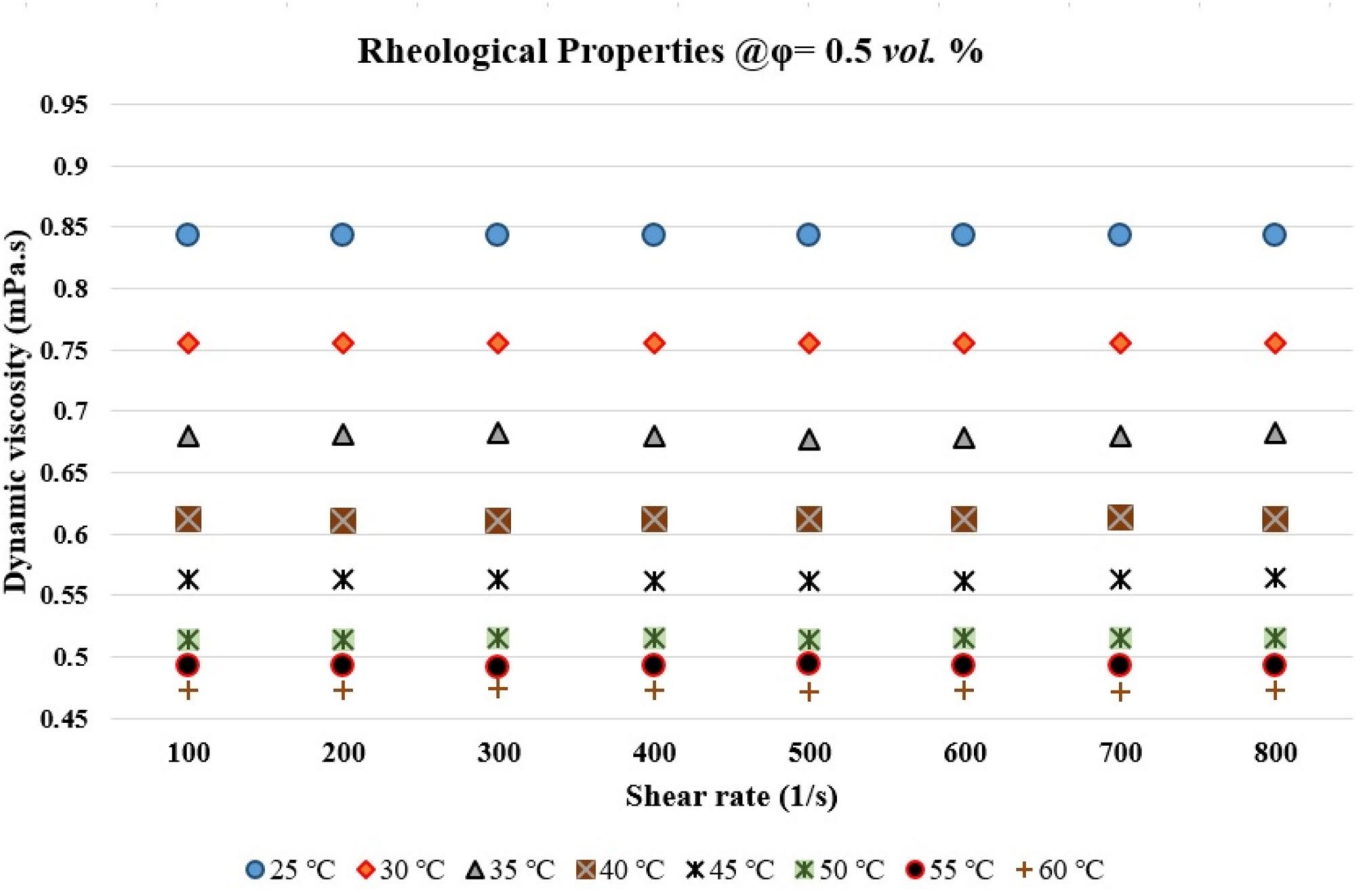
Rheological behavior of the MWCNT-water nanofluid: dynamic viscosity versus the shear rate at different temperatures and the solid concentration of 0.5 *vol.* %.

It is known that the rheological properties of nanofluids play a prominent role in dynamic viscosity and heat transfer. There are many debates on the rheological properties of the nanofluids containing carbon nanotubes (CNTs). While some researchers reported that CNT-based nanofluids show non-Newtonian behavior^46^, there are other researchers who observed that the CNT-based nanofluid with the solid concentrations less than 0.5 vol% show Newtonian behavior^47–49^, and increasing the solid concentration leads to changing the rheological behavior from Newtonian to non-Newtonian^50^. Thus, in the present study, the rheological properties of the studied nanofluid have been investigated over a different range of shear rates (100–800 1/s), temperatures, and solid concentration to realize that whether the studied nanofluid is a Newtonian or a non-Newtonian fluid. Figure 3 presents the results of dynamic viscosity variations versus shear rate in different temperatures and at the solid concentration of 0.5 vol%. As can be seen, the dynamic viscosity showed a linear trend in all the studied shear rates and temperature. Thus, it can be concluded that the studied nanofluid is a Newtonian fluid. The obtained results are in good agreement with the results presented by Halelfadel et al.^48^ and Afshari et al.^50^. It should also be noted that the dynamic viscosity versus shear rate showed the same trend in other studied solid concentrations.

### Dynamic viscosity

#### Effects of ultrasonication time on dynamic viscosity

**Figure 4:**
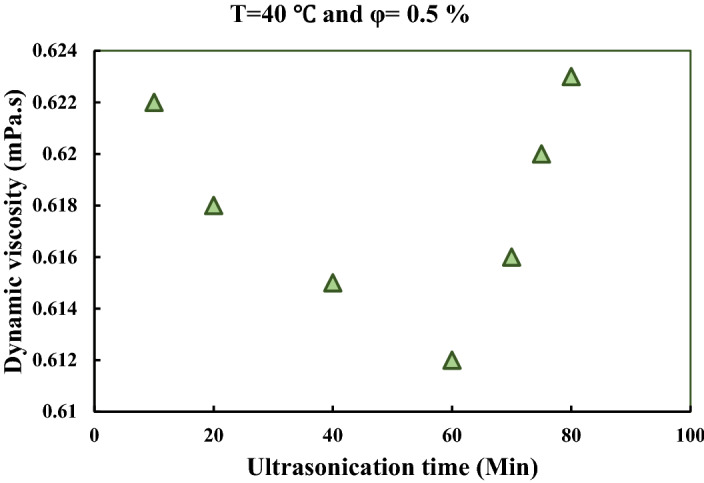
Effects of ultrasonication time on the variations of dynamic viscosity.

In order to investigate the effects of ultrasonication time on dynamic viscosity of MWCNT-water nanofluid, the dynamic viscosity of the samples has been measured over different ultrasonication times on the 30^th^ day after samples preparation, and the results have been presented in Fig. 4. This figure reveals that increasing the ultrasonication time leads to a gentle decrease in the dynamic viscosity of the samples. It would be because of the fact that increasing the sonication time leads to breaking down the MWCNT particles into the smaller size particles, which leads to the de-clustering of the MWCNT bundles. However, prolonging the ultrasonication time to higher than 60 min results in increasing the dynamic viscosity. Other researchers have been previously reported the same results indicating that there is an optimum ultrasonication time in which the dynamic viscosity reached its minimum value, and prolonging the ultrasonication time leads to increasing the dynamic viscosity^27,51^. It is also seen in Sect. 2.3 that 60 min ultrasonication leads to having a more uniform distribution of nanoparticles, which would be the main reason for having the minimum value of dynamic viscosity by 60 min ultrasonication. The effects of ultrasonication on the distribution of NPs and viscosity measurement would be better understood by the aid of Fig. IV (under the supporting information section).

#### Effects of temperature and solid concentration on dynamic viscosity

**Figure 5:**
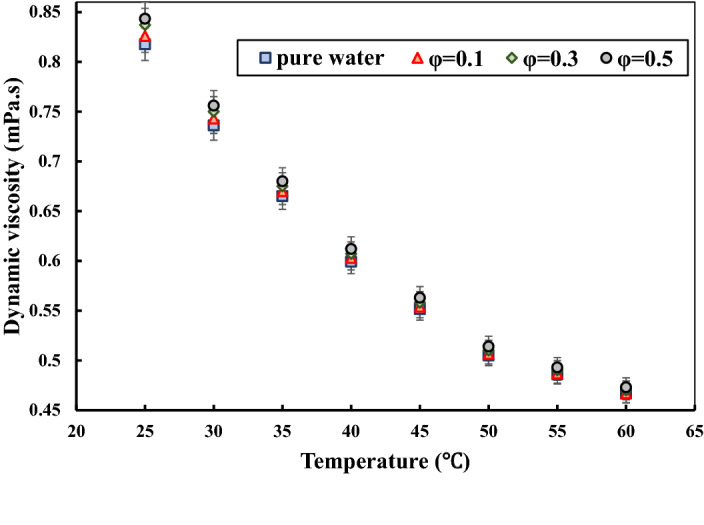
Variations of the dynamic viscosity of MWCNT-water nanofluid concerning temperature in different solid concentrations and at the ultrasonication time of 60 min.

After finding the optimum ultrasonication time in which the NF possesses the best stability and minimum dynamic viscosity, the dynamic viscosity of the MWCNT-water NF has been measured on different temperatures and solid concentrations, and at the ultrasonication time of 60 min. Figure 5 presents the variations of the dynamic viscosity with respect to temperature in the different studied solid concentrations. As can be seen, the dynamic viscosity showed a decreasing trend as the temperature started to rise. This trend is similar in all the solid concentrations. The main reason for this decrease would be that increasing the temperature leads to weakening the Van der Waals forces (inter-particles adhesion forces). It is interesting to note that the dynamic viscosity of the NF approached the dynamic viscosity of the base fluid in the temperatures higher than 45 °C. Moreover, it can be seen that increasing the solid concentration resulted in a slight increase in dynamic viscosity that is more noticeable in the temperatures of less than 40 °C. The maximum increase took place at the solid concentration of 0.5 vol% and the temperature of 25 °C by approximately 3%.

### Effects of adding MWCNT on pressure loss and pumping power management

The effects of adding MWCNT to the base fluid and employing ultrasonication, in different solid concentrations and temperatures, on the pressure loss will be discussed and presented in this section. It is known that the pressure loss, which is due to viscous effects, can be calculated as follows^52^:

1 $$\Delta P = f\frac{L}{D}\frac{{\rho V_{avg}^{2} }}{2}$$

where ***ρV***^***2***^_***avg***_***/2*** and ***f*** represent dynamic pressure and fanning friction factor, respectively. It must be noted that Eq. (1) is convenient to calculate the pressure loss for both the fully developed internal laminar and turbulent flow regimes^52^. The fanning friction factor can be calculated as follows^52^:

2 $$\begin{gathered} f_{la\min ar} = \frac{16}{{\text{Re}}} = \frac{{16\mu_{nf} }}{{\rho_{nf} VD}} \hfill \\ f_{turbulent} = 0.0791\left( {\frac{{\mu_{nf} }}{{\rho_{nf} VD}}} \right)^{0.25} \hfill \\ \end{gathered}$$

Moreover, ***ρ***_***nf***_ can be calculated, as presented by Pak and Cho^53^, as follows:

3 $$\rho _{{nf}} = \varphi \rho _{P} + (1 - \varphi )\rho _{{bf}}$$

where the indexes ***p*** and ***bf*** stand for particles and base fluid, respectively. It is known that adding NPs, which possess different density from the base fluid, to the base fluid affects the density of the resultant fluid (NF). Thus, in the present study, the density ratio is defined as the ratio of the NF’s density to that of the base fluid (water). Finally, investigating the effects of adding MWCNT to water on pressure loss, the fanning friction factor ratio (FFFR), which is the ratio of fanning friction factor of NF to that of the base fluid, is calculated for both the internal laminar and turbulent flow regimes^54^:

4 $$\begin{gathered} FFFR_{l\min ar} = \frac{{f_{nf} }}{{f_{water} }} = \left( {\frac{{\rho_{water} }}{{\rho_{nf} }}} \right)\left( {\frac{{\mu_{nf} }}{{\mu_{water} }}} \right) \hfill \\ FFFR_{turbulent} = \frac{{f_{nf} }}{{f_{water} }} = \left[ {\left( {\frac{{\rho_{water} }}{{\rho_{nf} }}} \right)\left( {\frac{{\mu_{nf} }}{{\mu_{water} }}} \right)} \right]^{0.25} \hfill \\ \end{gathered}$$

**Figure 6:**
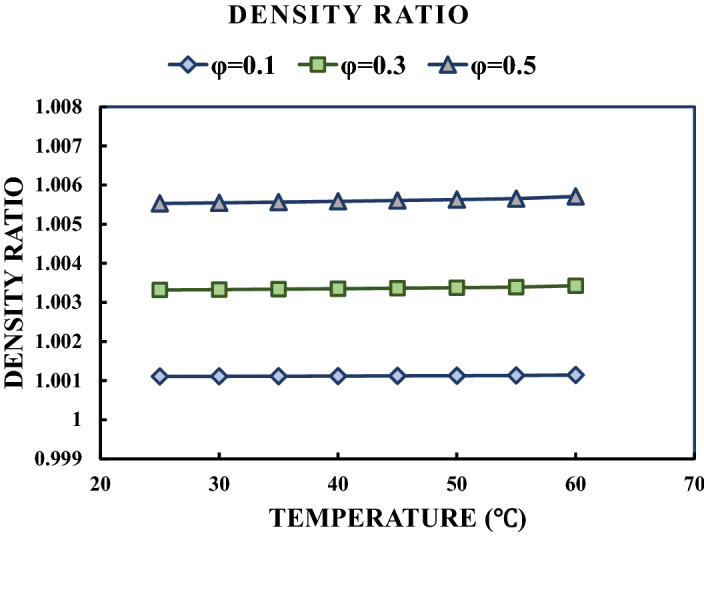
Variations of density ratio with respect to temperature in different solid concentrations.

**Figure 7:**
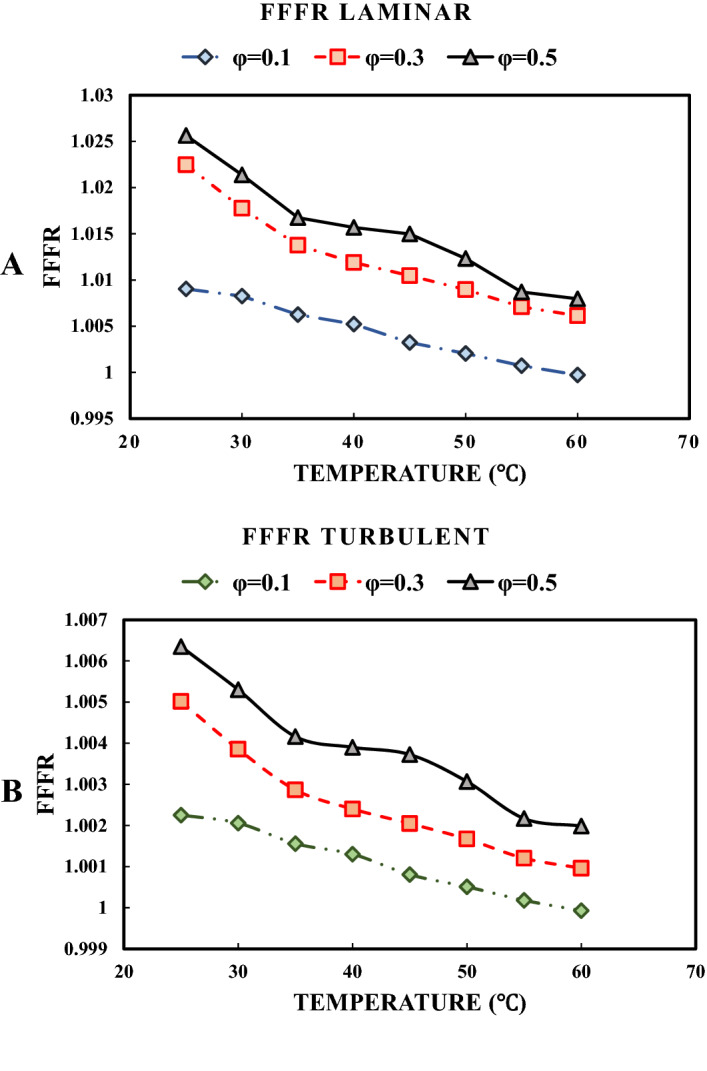
Variations of fanning friction factor ratio concerning temperature in different solid concentrations; (**A**) in a laminar flow, and (**B**) in a turbulent flow.

The variations of the density ratio with respect to temperature in different studied solid concentrations are presented in Fig. 6. It can be seen that the maximum increase in the density is just under 0.6%, which means that the increase in head loss and pumping power is negligible. Figure 7 presents the variations of FFFR with respect to temperature in different solid concentrations. As can be seen, adding MWCNT to water leads to increasing the FFFR in both the laminar and turbulent flow regimes. The maximum increase in FFFR for the laminar flow is just under 2.5%, while it is less than 0.7% for the turbulent flow regime. Moreover, increasing the temperature results in decreasing the FFFR for both the laminar and turbulent flow regimes. Thus, it can be concluded that using the MWCNT-water NF in the studied range of solid concentrations will not impose a considerable cost of pressure loss and pumping power.

## Concluding remarks

In the present study, the effects of ultrasonication time on the stability, rheological properties, and pumping power management of MWCNT-water nanofluid have been studied over different ranges of temperature and solid concentrations. The stability of the prepared samples has been scrutinized, employing the visual observation method and zeta potential analysis throughout 30 days after preparation. It is found that the optimum ultrasonication time in which the samples showed good stability is 60 min, and prolonging the ultrasonication time leads to deteriorating the stability of the samples. After that, using a Brookfield cone and plate viscometer (CAP2000 +), the viscosity of the samples has been measured in different temperatures and solid concentrations. The rheological behavior of the nanofluid has been studied, and it is found that the prepared nanofluid is a Newtonian fluid. It is also revealed that adding nanoparticles to the base fluid results in increasing the dynamic viscosity of the base fluid. However, the increase in dynamic viscosity by increasing the solid concentration was negligible, and the maximum increase took place at the solid concentration of 0.5 vol% and temperature of 25 °C by just higher than 3%. It is also observed that the dynamic viscosity of the nanofluid approached the dynamic viscosity of the base fluid in the temperatures higher than 45 °C. The effects of adding MWCNT to the water on the pumping power management have also been investigated in both the laminar and turbulent flow regimes, and it is revealed that the nanofluid in the range of studied solid concentration will not impose a considerable cost for providing the required energy for pumping power. Thus, based on the dynamic viscosity of the studied nanofluid, which showed a negligible increase, it would be a good idea for future works to investigate the thermal and fluid dynamic performance of the nanofluid as well as the heat transfer performance in a practical application.

## Supplementary information

Supplementary information.
