# Supplementary material for: Effects of ultrasonication time on stability, dynamic viscosity, and pumping power management of MWCNT-water nanofluid: an experimental study
Source: Sci Rep. 2020 Sep 16;10:15182. doi: 10.1038/s41598-020-71978-9 (PMC7494922; doi:10.1038/s41598-020-71978-9)
Supplement: Supplementary file 1 — Supplementary information. [file 41598_2020_71978_MOESM1_ESM.pdf]

Effects of Ultrasonication Time on Stability, Dynamic Viscosity, and Pumping Power Management of MWCNT-Water Nanofluid: An Experimental Study

Amin Asadi[1,2*], Ibrahim M. Alarifi[3,4]

[1]Institute of Research and Development, Duy Tan University, Da Nang 550000, Vietnam.
[2]Faculty of Natural Sciences, Duy Tan University, Da Nang 550000, Vietnam.
[3]Department of Mechanical and Industrial Engineering, College of Engineering, Majmaah University, Al-Majmaah 11952, Riyadh, Saudi Arabia
[4]Engineering and Applied Science Research Center, Majmaah University, Al-Majmaah 11952, Riyadh, Saudi Arabia
***Corresponding author**: aminasadi@duytan.edu.vn

Supporting information:

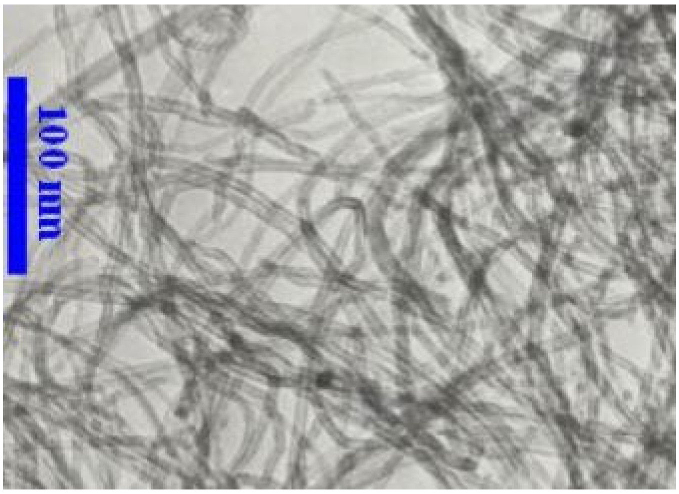

Fig. I. TEM image of MWCNT particles [55] **(Reprinted with the permission of Elsevier).**

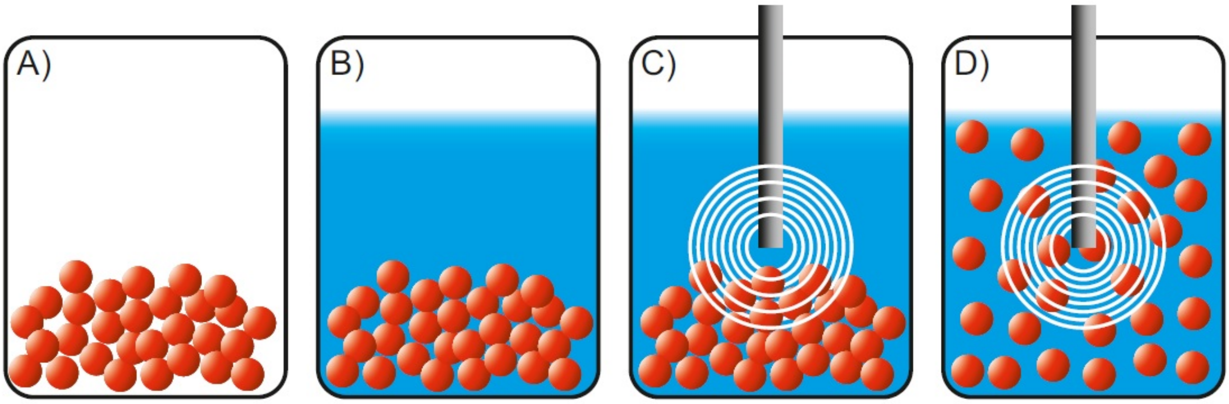

Fig. II. A schematic view of different stages of the two-step method [56] **(Reprinted with the permission of Elsevier).**

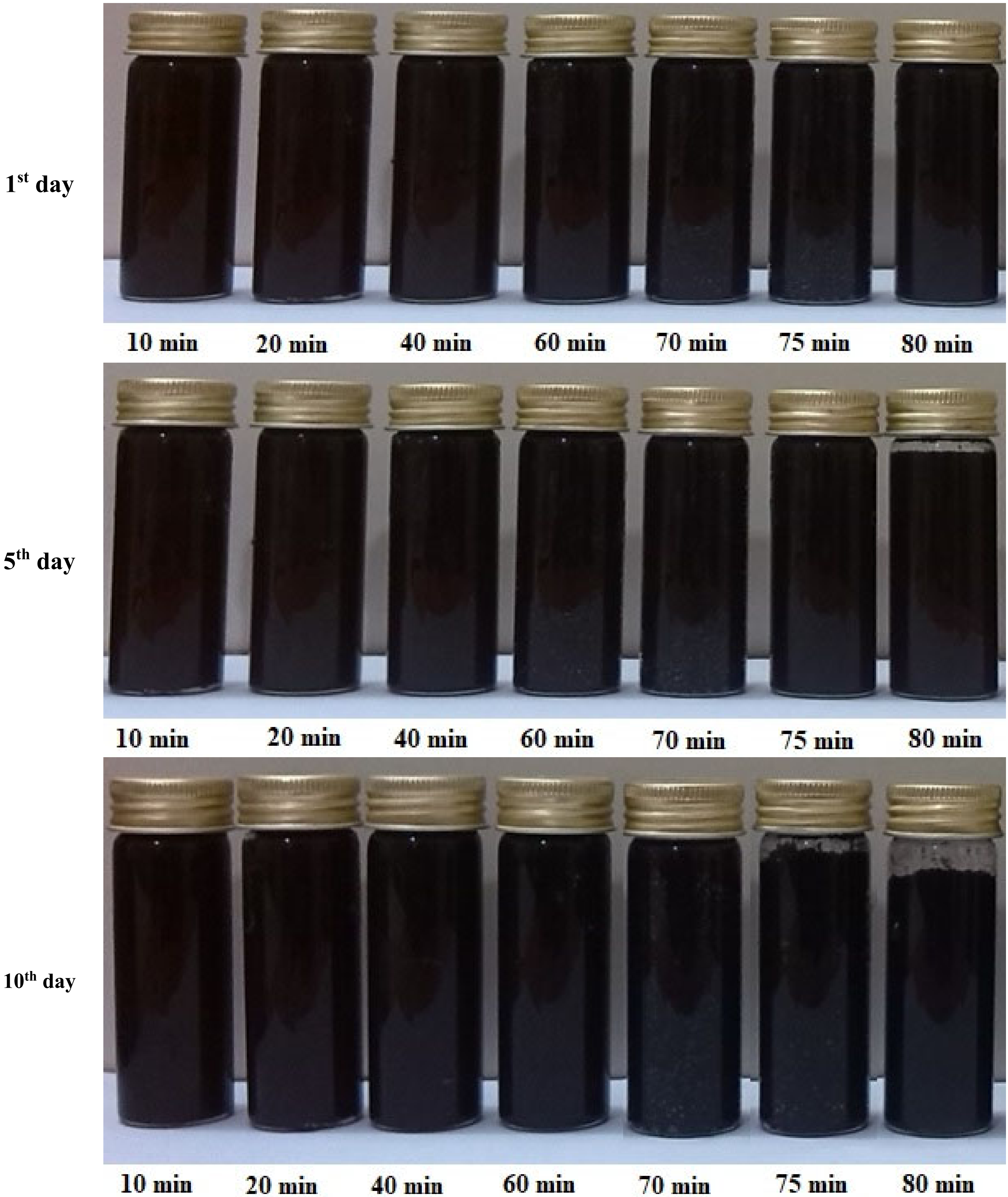

1st day
10 min
20 min
40 min
60 min
70 min
75 min
80 min
5th day
10 min
20 min
40 min
60 min
70 min
75 min
80 min
10th day
10 min
20 min
40 min
60 min
70 min
75 min
80 min

30th day

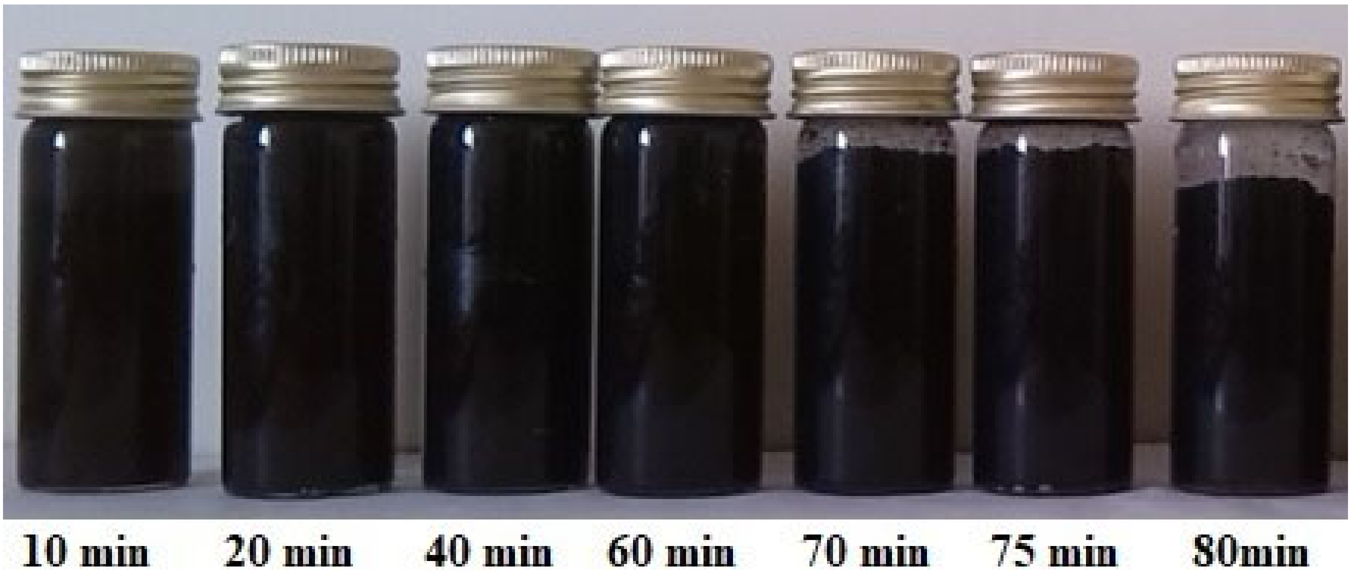

Fig. III. Stability analysis of the NF samples through visual observation over 30 days after preparation [38] **(Reprinted with the permission of Elsevier).**

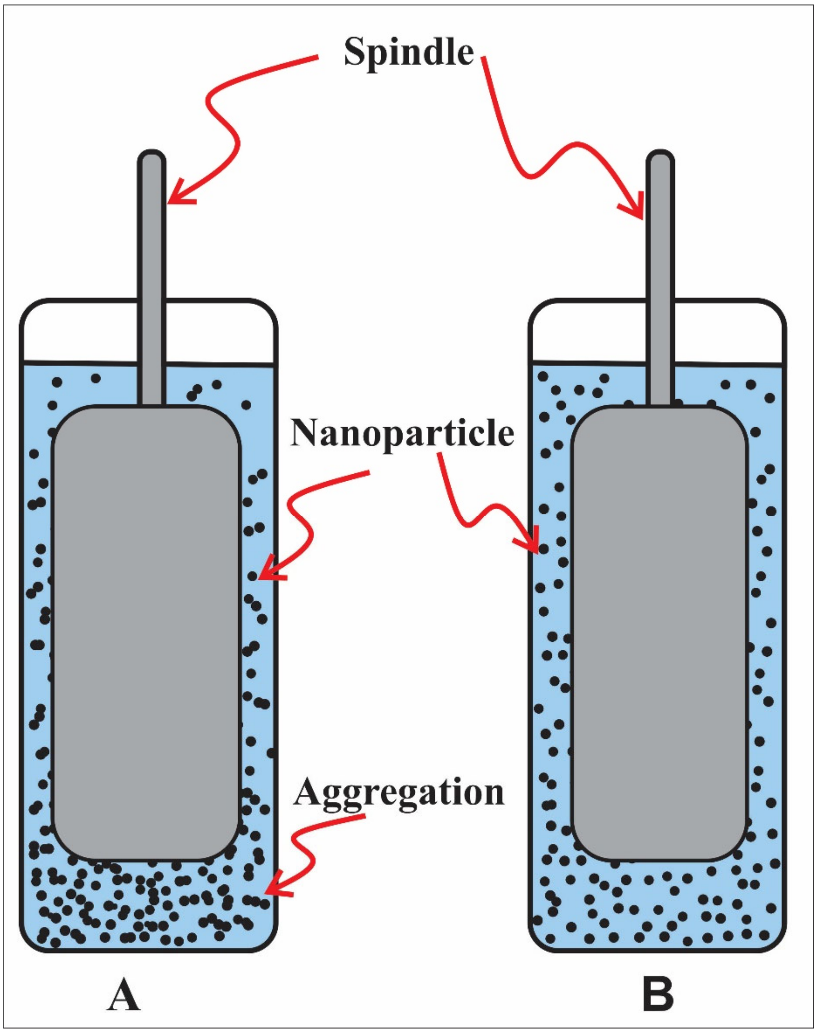

Fig. IV. A schematic view of the effect of ultrasonication on breaking down the cluster of particles and decreasing the viscosity; A) before ultrasonication, and B) after ultrasonication [36] **(Reprinted with the permission of Elsevier).**

Tab. I. Detailed information of MWCNT particles [55] **(Reprinted with the permission of Elsevier).**

**Outside diameter**	**<7 nm**
**Inside diameter**	**2-5 nm**
**Length**	**10-30 um**
**SSA**	**>500 m2/g**
**Electrical conductivity**	**>100 s/cm**
**True density**	**2.1 g/cm3**
**Purity**	**>95 wt %**
